# Trends in Breast Cancer Staging at Diagnosis Associated with Screening Campaigns in Lebanon

**DOI:** 10.1089/whr.2020.0076

**Published:** 2020-11-26

**Authors:** Christiane J. El Khoury, Salim M. Adib, Monique Chaaya, Khalil El Asmar, Maya Charafeddine, Nagi S. El-Saghir

**Affiliations:** ^1^ Department of Epidemiology and Population Health, Faculty of Health Sciences, American University of Beirut, Beirut, Lebanon.; ^2^ Department of Internal Medicine, American University of Beirut Medical Center, Beirut, Lebanon.

**Keywords:** outcomes, Arab, Middle-East, prevention

## Abstract

***Introduction:*** Mammography screening has been shown to improve early breast cancer (BC) detection, by shifting the disease at diagnosis to locally confined stages, offering lighter treatments and better prognoses. BC awareness campaigns calling for annual mammography screenings have been ongoing in Lebanon since 2002. Changes in BC staging at diagnosis as a consequence of documented improvements in mammography uptake remain to be described.

***Materials and Methods:*** We reviewed 2,822 BC cases identified by pathology reports in the American University of Beirut Medical Center between the years 1990 and 2015. After age stratification, we have trended the extracted stages versus time. Results were compared between the prescreening (1990–2001) and the postscreening period (2002–2015).

***Results:*** During the postscreening period, stage I represented 31%, stage II 47%, stage III 14%, and stage IV 8% of the cases. Stage I cases had more than doubled whereas stage III cases showed a mirror decrease compared with the years before the implementation of awareness campaigns. The increase in stage I was significantly more prominent in women aged 40 years and older (from 14% to 32%), compared with the younger group. Shifts in staging happened in parallel with a concurrent rise in reported uptake of mammography screening.

***Conclusions:*** Our findings demonstrate significant trends in earlier detection, which are likely associated with an increase in screening uptake and an awareness of BC as a public health issue. Staging data from hospitals all over Lebanon should be available for building national evidence. The Ministry of Public Health should require reporting of BC stage at diagnosis to the National Cancer Registry, as part of the annual cancer incidence reporting in Lebanon.

## Introduction

### Background and aim

Breast cancer (BC) is the most common malignancy in women worldwide and the second most common cancer overall.^1^ In Lebanon, BC incidence rates (IR) have been on the rise since the 1960s.^2^ Recent Lebanese BC data provided by the National Cancer Registry (NCR) indicated that in 2014, 2,528 new cases of primary BC were reported for all ages, accounting for 38% of all female cancers, and yielding an age-standardized IR of about 108.2 cases per 100,000.^3^ The median age at diagnosis among Lebanese women with BC has consistently been lower compared with Western countries. An analysis conducted by El-Saghir et al. in 2006 indicated that ever since 1998, the median age has hovered on a year-to-year basis around age 50, whereas it has been shown to be 61 years in the United States (2001–2005) and even 63 years in Western Europe.

In several Arab countries, the median age at diagnosis was found to be even younger than in Lebanon: 47 years in Saudi Arabia (2004), 45 in Kuwait (1993–1998), and 46 in Egypt (2001).^4,5^ The relatively younger median age at diagnosis in Lebanon and other Arab countries compared with Western ones has been attributed to the population pyramid skewed toward younger age groups in those countries rather than to a hypothetical genetic specificity.^5^

BC can be detected at early stages by using mammography.^6^ Early detection minimizes the physical and financial burden of the disease and improves prognosis.^7^ BC staging is based on TNM grading from the American Joint Committee on Cancer (AJCC).^8^ (Supplementary Appendix SA1).

Staging at diagnosis is an important factor of surveillance that illustrates the country's success at capturing early malignant disease.^7^ In a systematic review conducted by the World Health Organization (WHO) European region in 2017, it was recommended to maximize accurate and complete staging data in tumor registries since it played a distinctive role in the evaluation of BC control in low- to middle-income countries.^9^ Valid staging data are not currently available in the Lebanese NCR.^3^

Planned efforts to promote BC screening through regular mammography campaigns started in Lebanon in 2002. Before that date, screening occurred only sporadically in medical practice and its occurrence had never been documented. Initially, the annual campaigns were confined to the international BC month in October of every year. Since 2006, the annual campaigns have been expanded to cover all three autumn months, during which mammography is available free of charge at public centers, and at a minimal cost in participating private centers.

The Lebanese Ministry of Public Health (MOPH) published national recommendations in 2009, based on accumulated epidemiological data. They called for annual mammography for women with no personal or family history of BC to start at age 40, and to be repeated annually, for as long as a woman is in good health.^10^

**FIG. 1.**
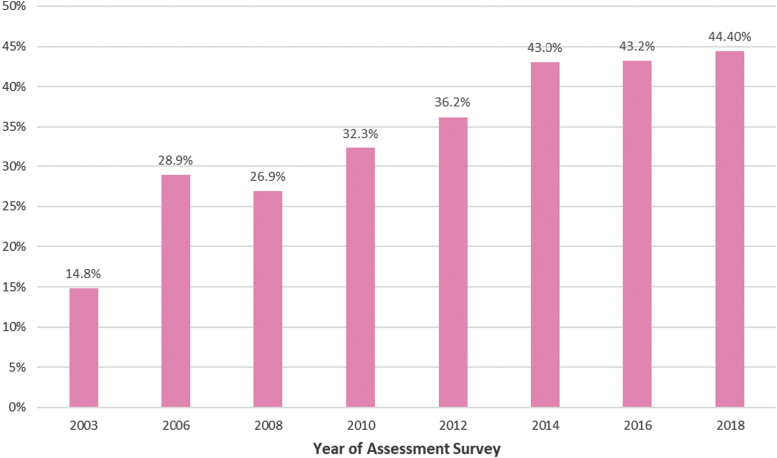
Percentage of first-time mammography uptake in Lebanese women (serial cross-sectional surveys of 1,200 randomly selected women 40 and older across Lebanon between 2003 and 2018)—source: Adib SM. Serial cross-sectional assessment of mammography uptake. Data partially published.

The first utilization of mammography increased slightly between 2002 and 2005 (from 11% to 18%), mainly among women aged between 40 and 49 compared with older age groups, and more among those residing in the Greater-Beirut (GB) area surrounding the capital city compared with those residing elsewhere.^11^ By 2014, the first-time use of mammography had reached about 45%, mostly among women in their 50s and those of a higher socioeconomic status.^12^ Periodic national sample surveys have shown that mammography uptake has been increasing gradually since then (Fig. 1).

Fifteen years since this campaign was launched, it has now become important to evaluate the impact of the screening campaigns on the status of BC staging at diagnosis in Lebanon. Changes in staging at diagnosis, if present, may be attributed to the screening campaigns, as more than half of 1,200 women surveyed serially between 2002 and 2005 consistently indicate that they had heard about the campaigns and were prompted to act as a result.^13^

Data on staging at diagnosis are still not available at the NCR, the evaluation, and subsequent cost-benefit analysis (CBA) of mammography screening, which should inform the future decisions of health policymakers, and have to be obtained indirectly from hospital-based sources. This article has performed a historical analysis of BC staging at diagnosis, from the pre-campaign era (1990–2001), up until the year 2015 by using data from the largest hospital in Lebanon, to generate new evidence that would eventually lead to a comprehensive CBA.

### Staging at diagnosis in the pre-campaign period in Lebanon

The first and only assessment of historical changes in BC staging at diagnosis in Lebanon before the initiation of the regular screening programs was conducted by Saghir et al. in 2006 on data derived from cases diagnosed from 1991 to 2001, at the American University of Beirut Medical Center (AUBMC) in Beirut. Visible trends started to appear in the early 2000s, showing proportions of earlier stages (I and II) rising whereas those of advanced stages (III and IV) were decreasing, most likely due to increased awareness about symptoms and lower stigma related to cancer diagnosis among younger, better educated women.^4^

In 2015, Chahine et al. examined the characteristics of BC among a smaller case series of Lebanese women diagnosed and treated at Hotel-Dieu de France (HDF) hospital in Beirut from 1990 until 2013. Aggregate data showed that BC staging at diagnosis had, indeed, improved compared with two periods before and after 2002.^14^

### Objectives

The objectives of this article were to measure the proportions of stages I and II (less advanced disease) compared with stages III and IV (advanced disease) at the time of diagnosis within a case series accumulated at the AUBMC between 1990 and 2015; and to establish a historical correlation with the uptake of BC screening available from concomitant serial surveys. Trends in BC staging by age groups at the time of diagnosis were also examined.

## Materials and Methods

### Study design and data sources

This historical correlational description draws data from de-identified hospital records of all cases of Lebanese female BC cases having received positive pathology results at AUBMC from 2000 to 2015. Parts of the analysis were supplemented by de-identified cumulative data from the previous historical analysis, covering years 1990–2001.^4^ Staging at diagnosis was not always directly available in reviewed records, and it had to be constructed by using clinical and pathological information available.

### Definition of variables

The main outcome extracted for this data analysis was the AJCC-derived staging at diagnosis (Supplementary Appendix SA1). Tumor size, the first component of the TNM staging, was also considered as a separate outcome, in view of its importance in increasing the probability of diagnosis. Outcomes were stratified by age at diagnosis, and they were divided in two age groups, based on the recommended age for initiation of mammography in Lebanon:

(1) Women diagnosed at age <40 years
(2) Women diagnosed at age 40 years and older

### Statistical analysis

The relative proportion of each of the four BC stages, the mean ages at diagnosis were tabulated, and their association was assessed. Percentages of stages were then plotted independently to assess the significance of their historical changes, starting from data collected in 1990. Different outcome proportions were compared from the period before 2002 and afterward, in correlation with available national figures of mammography uptake. Differences in proportions were tested by using chi-square test. Mean age differences before and after the start of the campaigns were tested by using the *t*-test.

Linear regression analysis was carried out to analyze the different trends across time. A *p*-value <0.05 was considered significant. All data were analyzed by using STATA version 13. Finally, to enhance generalizability of the findings, our results were compared with published data from a study performed in another Lebanese hospital.^11^

### Ethical considerations

In this analysis, the privacy of subjects and confidentiality of data were preserved through the de-identification of the medical records, and patients were not directly contacted. This study received an exemption as a secondary data analysis from the biomedical Institutional Review Board (IRB) in AUB.

## Results

Our study population consisted of 2,822 Lebanese female BC cases having received positive pathology results at AUBMC from 2000 to 2015. Of all the cases, 255 cases (9%) were excluded from the analysis because of incomplete staging data.

The mean age at diagnosis of all studied cases was 52 years (standard deviation [SD] ±12.6), whereas the median age was 50 years. When stratified over the pre- and postscreening periods, the mean age was 53.0 years (SD ±12.8) and 50.7 (SD ±12.3), respectively, with a significant change in mean age between both periods (*p* < 0.01).

Differences in tumor size at diagnosis were significant between the two periods studied. Tumor sizes <2 cm increased from 19% during the prescreening period to half of the cases during the postscreening period (*p* < 0.01). Conversely, tumors that were larger than 2 cm decreased significantly with time. Tumors between 2 and 5 cm decreased from 66% to 42%, whereas tumors larger than 5 cm in size decreased from 15% to 8%.

There were also significant historical differences in lymph node involvement from 64% to 44%. Even though the majority of cases were free of distant metastasis in both periods, metastatic case proportions increased slightly from 6% to 8%, moving from the prescreening to the postscreening period (*p* = 0.032).

Thus, BC staging at diagnosis changed over time. The relative proportion of stage I significantly increased from 14% to 31% (*p* < 0.01). Stage III demonstrated a significant mirror decrease between the two periods, from 35% to 14%. The proportion of stage II remained relatively stable over the two periods (45% up to 47%, respectively; *p* = 0.261). The proportion of the more advanced stage IV increased slightly but significantly from about 6% in the first period to about 8% in the more recent one (*p* = 0.032). Details are presented in Table 1.

**Table 1.** Relative proportions of female breast cancer staging characteristics at diagnosis related to the initiation of the national screening program (American University of Beirut Medical Center, Beirut, Lebanon, 1990–2015) (*N* = 2,822)

	Prescreening period (1990–2001)	Postscreening period (2002–2015)	Total	p
Mean age at diagnosis (SD)	50.7 (±12.27)	53.0 (±12.80)	52.0 (±12.60)	**<0.01**
Tumor size, *n* (%)				
<2 cm	226 (18.98)	555 (50.04)	781 (33.96)	**<0.01**
Between 2 and 5 cm	792 (66.49)	461 (41.57)	1,253 (54.48)	**<0.01**
More than 5 cm	173 (14.53)	93 (8.39)	266 (11.56)	**<0.01**
Total	1,191 (100)	1,109 (100)	2,300 (100)	—
Lymph node involvement, *n* (%)				
Negative (N0)	426 (35.80)	613 (55.88)	1,039 (45.41)	**<0.01**
Positive (N1, N2, N3)	765 (64.20)	484 (44.12)	1,249 (54.59)	
Total	1,191 (100)	1,097 (100)	2,288 (100)	
Metastasis, *n* (%)				
No (M0)	1,199 (94.26)	1,193 (92.12)	2,392 (93.18)	**0.032**
Yes (M1)	73 (5.74)	102 (7.88)	175 (6.82)	
Total	1,272 (100)	1,295 (100)	2,567 (100)	
Stage, *n* (%)				
Stage I	180 (14.15)	395 (30.50)	575 (22.40)	**<0.01**
Stage II	572 (44.97)	611 (47.18)	1,183 (46.08)	0.261
Stage III	447 (35.14)	187 (14.44)	634 (24.70)	**<0.01**
Stage IV	73 (5.74)	102 (7.88)	175 (6.82)	**0.032**
Total	1,272 (100)	1,295 (100)	2,567 (100)	—

Totals do not always add up, because missing or unavailable metastatic cancers are immediately classified as stage IV.

Significant *p*-values are presented in bold.

SD, standard deviation.

**FIG. 2.**
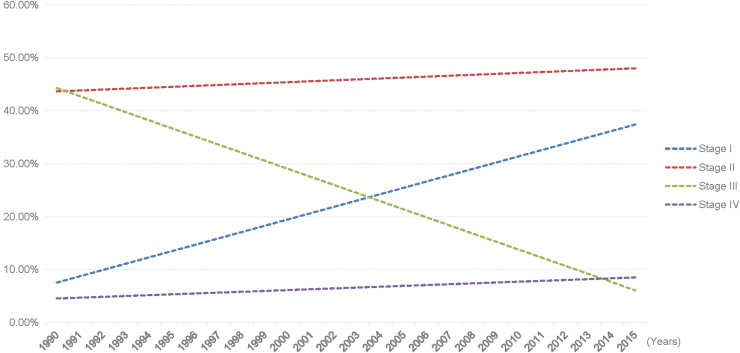
Linear trend of female BC stages at diagnosis in Lebanese women (from 1990 until 2015, *n* = 2,567). Stage I: *y* = 0.0119*x* + 0.0634 (95% CI: 0.009–0.015); *p* < 0.01. Stage II: *y* = 0.0017*x* + 0.4348 (95% CI: −0.002 to 0.0055); *p* = 0.34. Stage III: *y* = −0.0153*x* + 0.4581 (95% CI: −0.0191 to −0.0115); *p* < 0.01. Stage IV: *y* = 0.0016*x* + 0.0436 (95% CI: 0.0001 to 0.003); *p* = 0.034. BC, breast cancer; CI, confidence interval.

Figure 2 shows the fitted linear regression for the yearly relative proportion of each BC stage since 1990. Stage I proportions showed increasing trends across time, whereas stage III proportions presented mirroring decreasing trends. Stage II regression line was relatively stable along the years. As for stage IV, the linear regression demonstrated a slight increase across the large period studied (Fig. 2).

Table 2 explores the association of age at diagnosis with staging severity, since BC screening recommendation relies primarily on age.

**Table 2.** Relative proportions of female breast cancer stages at diagnosis stratified by age related to the initiation of the national screening program (American University of Beirut Medical Center, Beirut, Lebanon, 1990–2015) (*N* = 2,567)

Age group	<40 years old, n (%)			≥40 years old, n (%)		
Time period	Prescreening period (1990–2001)	Postscreening period (2002–2015)	p	Prescreening period (1990–2001)	Postscreening period (2002–2015)	p
Stage I	38 (15.02)	37 (19.47)	0.216	142 (13.94)	358 (32.40)	**<0.01**
Stage II	115 (45.45)	100 (52.64)	0.135	457 (44.84)	511 (46.24)	0.519
Stage III	91 (35.97)	32 (16.84)	**<0.01**	356 (34.94)	155 (14.03)	**<0.01**
Stage IV	9 (3.56)	21 (11.05)	**0.002**	64 (6.28)	81 (7.33)	0.338
Total	253 (100)	190 (100)	**—**	1,019 (100)	1,105 (100)	**—**

N: Includes only cases for whom staging was available.

Significant *p*-values are presented in bold.

For women <40 years, stage I proportionally increased from 15% to 19% (*p* = 0.216), when comparing the two historical intervals. However, the increase of stage I was significant and more pronounced in the older age category, going up from 14% to 32% (*p* < 0.01). The relative proportions of stage II did not significantly fluctuate over time in both age categories, whereas stage III decreased significantly by around half in both. Moving from the period between 1990 and 2001 to the period between 2002 and 2015, stage IV increased from 4% to 11% in the younger age group (*p* < 0.01). For women aged 40 years and older, this proportion increased from 6% to 7% (*p* = 0.338). Details are presented in Table 2.

### Trends in BC stage correlated with mammography uptake

**FIG. 3.**
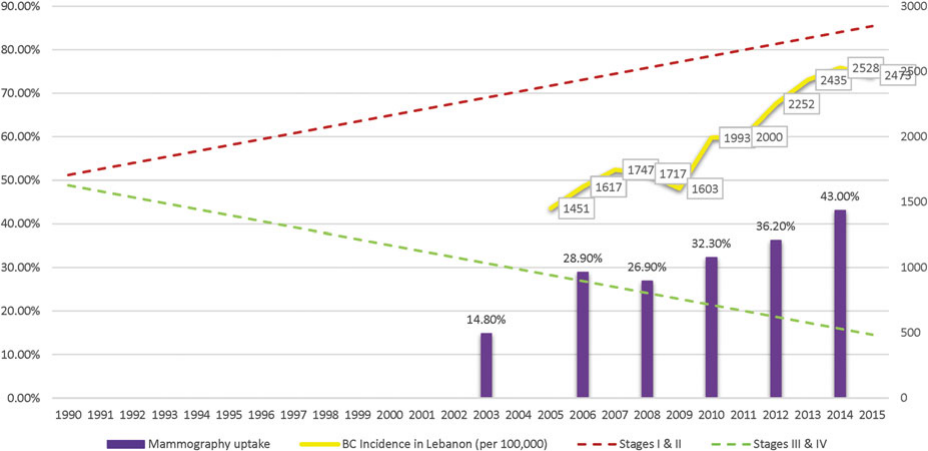
Correlation between linear trends in early (combined stages I and II) and advanced stages (combined stages III and IV) of female BC at diagnosis with reported uptake of mammography screening initiated as a national program in 2002 in parallel with BC incidence in Lebanon (from 1990 till 2015, *n* = 2,567). Stages I and II: *y* = 0.013*x* + 0.50 (95% CI: 0.009 to 0.017); *p* < 0.01. Stages III and IV: *y* = −0.013*x* + 0.50 (95% CI: −0.017 to −0.009); *p* < 0.01.

The linear regressions of combined early stage (stages I and II) and late-stage BC (stages III and IV) were plotted relative to data on the first uptake of mammography, which had increased gradually with time from 14.8% in 2003 to 43% in 2015. Stages I and II surged significantly upward across time whereas stages III and IV went downward. Details are presented in Figure 3.

## Discussion

BC incidence and caseload has been increasing year-to-year in Lebanon, suggesting an increase in BC diagnostic rates.^15^ Screening, established as a national program since 2002, is expected to catch cases at earlier stages of disease. Such an impact is strongly suggested in this correlational analysis.

Remarkable changes were detected in BC staging at diagnosis over the 26-year interval before and after the screening project started in 2002. Our findings show that over a 26-year period, the proportions of stage I have more than doubled from pre- to postscreening campaign periods. This increase was mirrored by a historical decrease in stage III proportions at diagnosis. Those trends were most significant after age 40, the age at which the first annual screening mammography is recommended in Lebanon for women with no family history of BC.

The move to earlier stages has affected all levels except stage IV. Results indicate that stage II has remained the most frequent one at diagnosis, and it has remained relatively stable across time. This apparent stability probably hides internal dynamics by which decreasing proportions of stage III had replaced increasing proportions of stage II, moving down to stage I. These internal dynamics in BC diagnosis over time toward less advanced stages can be suspected from studying changes in tumor size, the first and main component of the TNM classification. Results clearly indicated that the average tumor size at diagnosis had been moving steadily smaller, which is reflected in the down-staging phenomena.

The historical shift toward earlier stages found in the AUBMC series compares favorably with the results obtained from 612 BC patients analyzed as part of the HDF series between 1990 and 2013 (Supplementary Appendix SA2). In Table 3, trends of changes in proportions of BC stages over time are presented and appear as largely similar between the two Lebanese institutions.^14^

**Table 3.** Relative proportions of female breast cancer stages at diagnosis between pre-campaign and post-campaign screening periods (1990–2001 and 2002–2013) as reported in the two largest tertiary hospitals in Lebanon

n (%)	AUBMC^a^			HDF^b^		
Stage at diagnosis	Prescreening period (1990–2001)	Postscreening period (2002–2013)	p	Prescreening period (1990–2001)	Postscreening period (2002–2013)	p
Stage I	180 (15.01)	298 (31.20)	<**0.01**	39 (17.56)	83 (25.69)	**<0.01**
Stage II	572 (47.71)	525 (54.97)	**<0.01**	106 (47.75)	175 (54.18)	**0.04**
Stage III	447 (37.28)	132 (13.83)	**<0.01**	77 (34.69)	65 (20.13)	**<0.01**
Total^c^	1,199	955	—	222	323	—

^a^ American University of Beirut Medical Center (present findings).

^b^ HDF in Beirut.^11^

^c^ Stage IV was excluded from both groups, as it was an exclusion criterion in the HDF study.

Significant *p*-values are presented in bold.

AUBMC, American University of Beirut Medical Center; HDF, Hotel-Dieu de France.

Trends in staging over the 26-year interval considered in this analysis can be attributed directly and indirectly to the screening uptake. The increase in mammography uptake reaching around half of the Lebanese women in recent years can be directly linked to cancer down-staging. Indirectly, the annual message on BC screening may have increased the alertness in women and broken the fear associated with cancer diagnosis, thus contributing to earlier detection. At any rate, as the impact of the annual screening campaigns increases across ages and regions in Lebanon, favorable trends in early diagnosis should be sustained.

Findings from this study may be artifacts, associated with improvement in diagnostic techniques over the 26-year span rather than with any other planned variable. However, the consistency of those trends over time and the absence of sudden short peaks argue against the potential effects of confounding factors and in favor of the validity of findings.

A more serious potential source of errors may be associated with the fact that 9% of the cases eligible for analysis in this article were found with no data on staging in their medical records. Should all the cases with missing data belong to one specific stage at diagnosis, this may cause a serious selection bias. Nevertheless, the conformity with findings from a concurrent analysis in Lebanon seems to suggest that there was no specific selection bias among cases with missing data.

Finally, it can be argued that cases at AUBMC may not be representative of the entire population of BC cases in Lebanon. Patients in AUBMC are specifically from an affluent socioeconomic status, and this can potentially result in bias. Arguably, the majority of cases are drawn from the GB area in and around the capital city, where awareness, accessibility to screening and treatment may differ from elsewhere. Nevertheless, based on NCR national BC figures, around 21% of the Lebanese cancer cases in 2010 and around 26% in 2014 were diagnosed and treated at AUBMC.^15^ It can be argued that a center treating one in every four cases in a small country such as Lebanon may actually be drawing a sample less severely biased than expected, throughout an active referral system. There are no reasons to believe that findings valid in 25% of cases in Lebanon would not represent the same trends for all Lebanese cases.

### Age and staging

In this analysis, the most advanced cases increased slightly over time. The increase was only significant in women younger than 40, in whom the disease is known to be notably more aggressive.^16^ As a consequence of the aging process of the Lebanese population, the age at diagnosis might be slowly shifting toward older age groups. This reinforces the argument for not recommending mammography to younger ages in Lebanon despite the severity of disease, as is argued by some practitioners. Lowering the recommended age for mammography initiation at a time of falling incidence would increase the rate of false-positive readings and add unneeded costs to the screening program.

The slight increase in stage IV can be largely explained by the fact that AUBMC, as an advanced tertiary hospital, often captures referred cases that are also the more complicated ones. Referred cases originate in majority from smaller centers in Lebanon, where late diagnosis may be more frequent, but this reality is not evidenced in this analysis or in any other relevant publication. One element suggestive of this attribution comprises findings showing that mammography uptake is stagnating in several areas away from GB.^11^

An age effect was also found in association with stage I increase over time. The sharpest increase was detected in the group of women eligible for screening (40 and above), as opposed to the younger group. Further, the stage I presence became significant among women aged older than 40 years during the screening period. These findings further indicate that screening recommendations calling for an annual mammography starting age 40 are being adopted by Lebanese women and their physicians, and these are resulting in visible benefits.

## Conclusions and Practical Implications

Although AUBMC is a national institution that captures a representative portion of the Lebanese BC cases, more data derived from other national institutions are needed to further support the information found on the improved stage at disease presentation. Staging data are not routinely collected by the Lebanese NCR, despite their importance in showing the effectiveness of the annual screening campaigns and related public health activities. The current analysis helps in compensating this gap and thus contributes to the CBA of BC screening needed to eventually update the national guidelines. The update is important to ensure adequate resource allocation to control the growing incidence of BC in Lebanon.

Stressing the importance of staging data and screening history at the national level will, in turn, promote the sustainability of CBA in Lebanon. The MOPH should request the reporting of staging at diagnosis to its NCR, as it adds to the completeness of the data and to the accuracy of our surveillance systems.

Our findings endorse the currently available screening efforts by demonstrating an improvement in BC staging in targeted age groups. National guidelines calling for annual mammography starting age 40 appear to be adequately followed and effective in the early detection in Lebanon. Ensuring that campaigns are sustained and increasingly more successful is one sure way of realizing further progress toward down-staging BC at diagnosis. Detailed analyses of mammography intake and stages at diagnosis should be de-accumulated to allow regional particularities to be detected and targeted for specific efforts.

## Supplementary Material

Supplemental data

Supplemental data

## Supplementary Material

Supplementary Appendix SA1

Supplementary Appendix SA2

## Abbreviations Used

AJCC: American Joint Committee on Cancer
AUBMC: American University of Beirut Medical Center
BC: breast cancer
CBA: cost-benefit analysis
CI: confidence interval
GB: Greater-Beirut
HDF: Hotel-Dieu de France
IRB: Institutional Review Board
NCR: National Cancer Registry
MOPH: Ministry of Public Health
SD: standard deviation
WHO: World Health Organization

## References

[1] Clegg LX, Reichman ME, Miller BA, et al. Impact of socioeconomic status on cancer incidence and stage at diagnosis: Selected findings from the surveillance, epidemiology, and end results: National Longitudinal Mortality Study. Cancer Causes Control 2009;20:417–435 19002764 10.1007/s10552-008-9256-0 PMC2711979

[2] Lakkis NA, Adib SM, Osman MH, Musharafieh UM, Hamadeh GN Breast cancer in Lebanon: Incidence and comparison to regional and Western countries. Cancer Epidemiol 2010;34:221–225 20413361 10.1016/j.canep.2010.02.013

[3] Shamseddine A, Saleh A, Charafeddine M, et al. Cancer trends in Lebanon: a review of incidence rates for the period of 2003–2008 and projections until 2018. Popul Health Metr 2014;12:4 24593777 10.1186/1478-7954-12-4 PMC3996020

[4] El Saghir NS, Seoud M, Khalil MK, et al. Effects of young age at presentation on survival in breast cancer. BMC Cancer 2006;6:194 16857060 10.1186/1471-2407-6-194 PMC1555600

[5] Shamseddine A. Breast cancer epidemiology in Lebanon. Breast Cancer. Human Health 2019:46–48

[6] El Saghir NS, Farhat RA, Charara RN, Khoury KE Enhancing cancer care in areas of limited resources: our next steps. Future Oncol 2014;10:1953–1965 25386812 10.2217/fon.14.124

[7] Rogers C IARC handbooks of cancer prevention, volume 7: Breast cancer screening. Public Health 2004;118:384

[8] Edge SB; American Joint Committee on Cancer, eds. AJCC cancer staging manual, 7th ed. New York: Springer, 2010:648 p. 10.1245/s10434-010-0985-4 20180029

[9] Altobelli E, Rapacchietta L, Angeletti PM, Barbante L, Profeta FV, Fagnano R Breast cancer screening programmes across the WHO European region: Differences among countries based on national income level. Int J Environ Res Public Health [Internet] 2017;14:452 10.3390/ijerph14040452 PMC5409652 28441745

[10] Adib SM, El Saghir NS, Ammar W Guidelines for breast cancer screening in Lebanon Public Health Communication. J Med Liban 2009;57:72–74 19623881

[11] Adib SM, Sabbah MA, Hlais S, Hanna P Research in action: mammography utilization following breast cancer awareness campaigns in Lebanon 2002-05. East Mediterr Health J 2009;15:6–18 19469422

[12] Elias N, Bou-Orm IR, Adib SM Patterns and determinants of mammography screening in Lebanese women. Prev Med Rep 2017;5:187–193 28070475 10.1016/j.pmedr.2016.12.015 PMC5219635

[13] Abi Abdallah Doumit M, Arevian M, Fares S Knowledge, attitude and practice of lebanese women towards breast cancer, breast self-examination and mammography. 2016 Available at: https://sigma.nursingrepository.org/handle/10755/602569 (accessed 1228, 2019)

[14] Chahine G, El Rassy E, Khazzaka A, et al. Characteristics of incident female breast cancer in Lebanon, 1990–2013: Descriptive study of 612 cases from a hospital tumor registry. Cancer Epidemiol 2015;39:303–306 25828075 10.1016/j.canep.2015.03.008

[15] National Cancer Registry (NCR). Lebanese Ministry of Public Health, moph [Internet]. Available at: http://www.moph.gov.lb (accessed 1228, 2019)

[16] El Chediak A, Alameddine RS, Hakim A, et al. Younger age is an independent predictor of worse prognosis among Lebanese nonmetastatic breast cancer patients: analysis of a prospective cohort. Breast Cancer (Dove Med Press) 2017;9:407–414 28670139 10.2147/BCTT.S130273 PMC5479304

