# Supplementary material for: Trends in Breast Cancer Staging at Diagnosis Associated with Screening Campaigns in Lebanon
Source: Womens Health Rep (New Rochelle). 2020 Nov 26;1(1):521–8. doi: 10.1089/whr.2020.0076 (PMC7785066; doi:10.1089/whr.2020.0076)
Supplement: Supplemental data [file Supp_AppendixSA1.docx]

APpENDIX 1

BREAST CANCER STAGING

Tumor stage is determined by the size of the tumor, number of involved lymph nodes, and whether the cancer has spread to other parts of the body. The TNM staging system classifies cancers based on their T, N, and M stages:

• The letter T followed by a number from 0 to 4 describes the tumor’s size and spread to the skin or to the chest wall under the breast. Higher T numbers mean a larger tumor and/or wider spread to tissues near the breast.

• The letter N followed by a number from 0 to 3 indicates whether the cancer has spread to lymph nodes near the breast and, if so, how many lymph nodes are affected.

• The letter M followed by a 0 or 1 indicates whether the cancer has spread to distant organs for example, the lungs or bones.^1^

Once the T, N, and M categories have been determined, this information is combined in a process called stage grouping, as shown below. Cancers with similar stages tend to have a similar outlook and are often treated in a similar way. The stage is expressed in Roman numerals from stage 0 to stage IV. Stage 0 breast cancer (i.e., in situ breast cancer) is characterized by an accumulation of malignant cells that have not invaded into surrounding tissue. Breast tumors designated as stage I, II, III, or IV involve some invasion of tumor cells beyond the basement membrane, and are thus referred to as invasive tumors.^1^

TNM and stages adapted from the AJCC staging companion ^1^


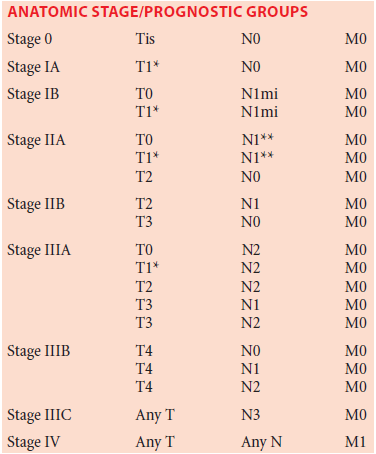


REFERENCES

1. Edge SB, American Joint Committee on Cancer, editors. AJCC cancer staging manual. 7th ed. New York: Springer; 2010. 648 p.
