# Supplementary material for: Trends in Breast Cancer Staging at Diagnosis Associated with Screening Campaigns in Lebanon
Source: Womens Health Rep (New Rochelle). 2020 Nov 26;1(1):521–8. doi: 10.1089/whr.2020.0076 (PMC7785066; doi:10.1089/whr.2020.0076)
Supplement: Supplemental data [file Supp_AppendixSA2.docx]

APpENDIX 2

BREAST CANCER STAGE COMPARISON BETWEEN TWO LARGEST TERTIARY HOSPITALS IN LEBANON

Table 1: Relative proportions of female breast cancer stages at diagnosis between pre-campaign and post-campaign screening periods (1990 – 2001 and 2002 – 2013) as reported in the two largest tertiary hospitals in Lebanon

| **n (%)** | **AUBMC*** | | | **HDF**** | | |
| --- | --- | --- | --- | --- | --- | --- |
| **Stage at diagnosis** | **Pre-screening period**  **(1990 – 2001)** | **Post-screening period**  **(2002 – 2013)** | **P-value** | **Pre-screening period**  **(1990 – 2001)** | **Post-screening period**  **(2002 – 2013)** | **P-value** |
| **Stage I** | 180 (15.01) | 298 (31.20) | **<0.01** | 39 (17.56) | 83 (25.69) | **<0.01** |
| **Stage II** | 572 (47.71) | 525 (54.97) | **<0.01** | 106 (47.75) | 175 (54.18) | **0.04** |
| **Stage III** | 447 (37.28) | 132 (13.83) | **<0.01** | 77 (34.69) | 65 (20.13) | **<0.01** |
| **Total***** | 1199 | 955 | - | 222 | 323 | - |

- American University of Beirut Medical Center (present findings)

** Hôtel-Dieu de France in Beirut ^11^

*** Stage IV was excluded from both groups, as it was an exclusion criterion in the HDF study
